# High-Efficiency CRISPR/Cas9-Mediated Correction of a Homozygous Mutation in Achromatopsia-Patient-Derived iPSCs

**DOI:** 10.3390/ijms24043655

**Published:** 2023-02-11

**Authors:** Laura Siles, Paula Gaudó, Esther Pomares

**Affiliations:** 1 Fundació de Recerca de l’Institut de Microcirurgia Ocular, 08035 Barcelona, Spain; 2 Departament de Genètica, IMO Grupo Miranza, 08035 Barcelona, Spain

**Keywords:** CRISPR/Cas9, gene editing, achromatopsia, inherited retinal dystrophies

## Abstract

Achromatopsia is an autosomal recessive disorder, in which cone photoreceptors undergo progressive degeneration, causing color blindness and poor visual acuity, among other significant eye affectations. It belongs to a group of inherited retinal dystrophies that currently have no treatment. Although functional improvements have been reported in several ongoing gene therapy studies, more efforts and research should be carried out to enhance their clinical application. In recent years, genome editing has arisen as one of the most promising tools for personalized medicine. In this study, we aimed to correct a homozygous *PDE6C* pathogenic variant in hiPSCs derived from a patient affected by achromatopsia through CRISPR/Cas9 and TALENs technologies. Here, we demonstrate high efficiency in gene editing by CRISPR/Cas9 but not with TALENs approximation. Despite a few of the edited clones displaying heterozygous on-target defects, the proportion of corrected clones with a potentially restored wild-type PDE6C protein was more than half of the total clones analyzed. In addition, none of them presented off-target aberrations. These results significantly contribute to advances in single-nucleotide gene editing and the development of future strategies for the treatment of achromatopsia.

## 1. Introduction

Achromatopsia (ACHM) is an inherited retinal dystrophy (IRD) affecting approximately 1 in every 30,000 individuals [1,2]. It is an autosomal-recessive disorder associated with a loss of cone photoreceptor function [1,2,3,4]. ACHM usually shows an early onset presenting pendular nystagmus, poor visual acuity, lack of color vision, and photophobia [2,3,5]. Patients with a milder form of visual acuity and presenting residual color vision are classified as incomplete achromats [2,3,5]. Few genes have been associated with ACHM pathogenesis, all of them encoding cone-phototransduction proteins or involved in their development (*CNGA3*, *CNGB3*, *GNAT2*, *PDE6C*, *PDE6H,* and *ATF6*) [4,5,6]. Notably, the vast majority of reported damaging variants in these genes are point mutations, which primarily comprise missense and nonsense changes [1,2,7,8,9].

Retinal degeneration in IRDs can occur due to defects in the phototransduction cascade or other pathways related to retinal cell function [10]. Several pathogenic variants involved in cone survival, retina neurotransmission [10], and vascularization [11,12] are reportedly associated with photoreceptor dystrophies. For example, oxidative stress leads to alterations in extracellular matrix-mediated signaling by inducing apoptosis [13]. Additionally, *ATF6* has been postulated to link endoplasmic reticulum stress with photoreceptor damage and apoptosis in ACHM [14].

Phosphodiesterase 6C (*PDE6C*, [OMIM 600827]), which is specifically expressed in cones, is a component of the 3’,5’-cyclic nucleotide phosphodiesterases group (PDEs). *PDE6C* is located in chromosome 10 (q23.33) and comprises 22 exons codifying an 858 amino acid protein [15]. PDE6C participates in signal transduction by controlling cellular levels of cGMP upon the light excitation of cone visual-pigment molecules [1,5]. Specifically, the PDE6 complex in cone photoreceptors is composed of two catalytic subunits of PDE6C and two regulatory subunits of PDE6H [16]. Following light stimulation, PDE6C becomes activated and hydrolyzes intracellular cGMP molecules resulting in hyperpolarization of the photoreceptor plasma membrane [7]. All components of the PDE group contain a catalytic core (PDEase domain) of approximately 270 amino acids, which is regulated by flanking regulatory domains [17,18]. 

In the last few decades, the discovery and development of gene editing tools have revolutionized the era of personalized medicine [19]. CRISPR/Cas nucleases, together with transcription activator-like effector nucleases (TALENs), are the most commonly used strategies for DNA engineering. Advances in these technologies directly impact disease treatment, modeling, and diagnosis [20,21]. Both TALENs and CRISPR/Cas systems target specific DNA through engineered guides. Specifically, TALENs are synthesized by fusing the catalytic domain of the restriction endonuclease FokI to the C-terminus of the TALE sequence by which DNA is recognized. TALENs work in pairs, binding both DNA strands in opposite orientations so that FokI can dimerize and cleave DNA between the two TALENs [16,22,23]. On the other hand, CRISPR/Cas uses a guide RNA (sgRNA) complementary to the target DNA that must, necessarily, be next to a protospacer adjacent motif (PAM) required by Cas endonuclease to cut the DNA. After DNA double-strand breaks (DSBs), cellular machinery primarily repairs DNA via either non-homologous end-joining (NHEJ) or homology-directed repair (HDR) in cases where a donor repair template is provided [16,24]. Notably, HDR is less efficient and is not commonly employed by dividing cells if compared with random repair processes, such as NHEJ or microhomology-mediated end joining (MMEJ) in mammalian cells [25,26,27,28,29,30]. 

Unfortunately, there are still no proven treatments to cure IRDs; therefore, genetic testing and counseling are crucial aspects oftheir management [2]. Refractive correction and other approaches to improve visual quality are also commonly utilized to reduce patients’ symptomatology [2]. Consequently, there is an unmet need for developing therapeutic strategies to treat these disorders. Several clinical trials are ongoing for the treatment of Leber congenital amaurosis [31,32], retinitis pigmentosa [33], and corneal dystrophies [34], among others. Regarding ACHM, some studies aiming to restore *CNGA3* and *CNGB3* expression by gene replacement are in progress [35,36,37]. For instance, one has the objective of evaluating the long-term safety and efficacy of subretinal gene therapy on *CNGA3*-related ACHM [38]. Additionally, it is being explored whether the ciliary neurotrophic factor (CNTF) function, which protects cone photoreceptors from degeneration in several non-human models, could be translated to human ACHM patients carrying *CNGB3* mutations [37,39]. 

Gene and cell therapy represent promising strategies for next-generation medicine, with a special focus on pathologies caused by genetic defects [24]. In addition, genome editing constitutes a valuable tool for therapeutic applications, demonstrating striking progress in precision and efficiency [24]. However, despite the notable improvements achieved in recent years, gene therapy is not applicable to all diseases [24], whereas gene editing tools provide a huge therapeutic potential with the capability of virtually modifying any DNA sequence throughout the genome. Thus, gene editing has arisen as a viable approximation for genetic-related diseases because mutated DNA is corrected in situ. In the last years, in vivo CRISPR-based therapeutic strategies have been continuously growing [24,40,41]. The majority of human genetic diseases are caused by point mutations [25]; therefore, gene editing constitutes a promising and important tool for future precise and personalized medicine, and, importantly, for IRDs treatment. Nonetheless, several concerns regarding safety, delivery, and specificity are yet to be overcome [41].

In this study, we demonstrate the efficient correction of a homozygous *PDE6C* pathogenic variant in patient-derived iPSCs. We tested CRISPR/Cas9 and TALENs genome-editing technologies and achieved successful correction of the pathogenic variant using the CRISPR/Cas9 system without genomic alterations in the predicted off-targets analyzed. Nonetheless, we found few clones displaying on-target defects, but in all cases, these alterations were in heterozygosis. Significantly, more than half of the screened clones exhibited at least one corrected allele, suggesting a putative reversion of the pathogenic phenotype, because ACHM is an autosomal recessive disorder. Finally, gene editing did not compromise the expression of pluripotency markers in these corrected clones, nor their ability to differentiate into the three germ layers.

## 2. Results

### 2.1. Single-Nucleotide Gene Editing for Correcting a Homozygous PDE6C Variant Causing Achromatopsia

**Figure 1:**
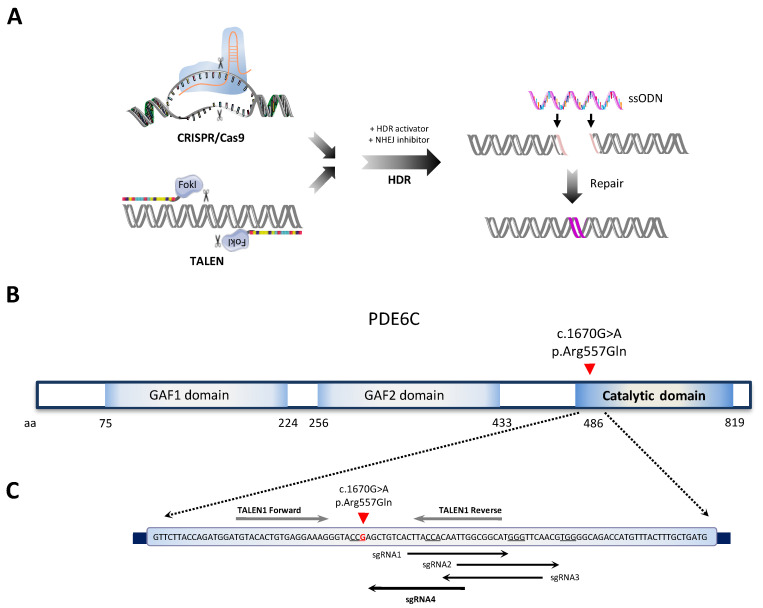
Single-nucleotide gene editing for the correction of a homozygous *PDE6C* variant causing achromatopsia. (**A**) Schematic of the assay followed for HDR-mediated gene editing by CRISPR/Cas9 and TALEN technologies. The figure was partially generated using Servier Medical Art, provided by Servier, licensed under a Creative Commons Attribution 3.0 unported license. (**B**) Representation of PDE6C protein domains. Homozygous patient’s mutation targeted for correction in exon 13 is depicted with a red arrowhead. (**C**) Schematic of the different TALEN and sgRNA guides designed for targeting the c.1670G>A mutation (nucleotide in red) on exon 13 of *PDE6C*. Arrows indicate sgRNA orientation. PAM sequences are underlined for each sgRNA.

According to the HGMD database, there are more than 70 variants in *PDE6C* described as damaging [42]. Single-base substitutions—missense, nonsense, and splicing-related—account for more than 85% of the *PDE6C* mutations. Conversely, small deletions, insertions, or duplications comprise only 13% of the pathogenic variants [42]. In an attempt to correct a human pathogenic missense *PDE6C* variant, we performed single-stranded oligodeoxynucleotide (ssODN)-mediated gene editing by CRISPR/Cas9 and TALEN technologies (Figure 1A).

For *PDE6C* editing, we used patient-derived iPSCs (the patient is hereafter referred to as Fi22/02, and the hiPS cell line as FRIMOi007-A), which carries a c.1670G>A (p.Arg557Gln) missense mutation in homozygosis (Figure 1B). This *PDE6C* variant—located in the catalytic domain—was predicted to be deleterious with a very low population frequency (rs201309785 and MAF = 0.00008) and affecting a highly conserved amino acid residue. 

Then, we screened this *PDE6C* locus for potential sgRNA and TALENs guides close to the pathogenic variant in exon 13 (Figure 1C). We identified four sgRNAs in the vicinity of the patient’s mutation, according to the presence of the canonical NGG PAM sequence—needed by Cas9 to produce DSBs—(underlined in Figure 1C). The shortest distance possible between the DSB and the nucleotide targeted for editing has been considered a key factor for sgRNA designs, together with the number of off-targets. Conversely, the TALEN mRNA pair was selected depending on the maximum predicted cleavage efficiency scored for targeting this genomic location.

To explore which of the sgRNAs would be most suitable for the gene editing assay, we analyzed all the characteristics mentioned above. As shown in Table 1, the distance between the DSB and editing site ranged between 3 and 35 nucleotides, and all four sgRNAs exhibited several off-targets (Table S1). sgRNAs from one to three displayed relatively large distances but have a low number of predicted off-targets (Table 1). Conversely, sgRNA4 has more off-targets but would apparently be the most appropriate guide for single-nucleotide editing according to its shortest distance (Table 1). Nonetheless, chromosomic location analysis from these off-targets revealed that none of the sgRNA4 off-targets were located in exonic regions. In contrast, sgRNA2 and sgRNA3 have one homologous sequence in a codifying region (Table 1), which, importantly, corresponds to exon 13 of *PDE6A*, another IRD-causative gene (Table S1) [43]. Notably, none of the sgRNAs have off-targets with fewer than three mismatches in sequence homology, making them all potential candidates for gene editing assays.

### 2.2. Selection of the Optimal sgRNA, TALEN, and ssODN Designs for Targeting the Pathogenic PDE6C Variant

**Figure 2:**
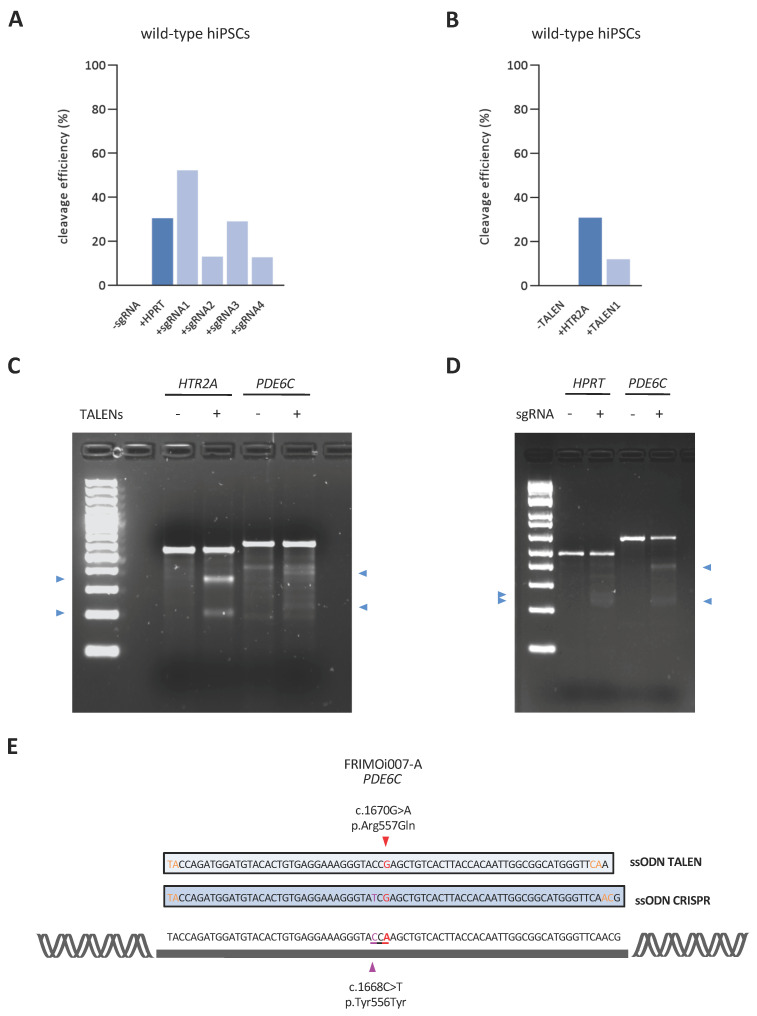
Selection of the optimal sgRNA, TALEN, and ssODN designs for targeting the pathogenic *PDE6C* variant. (**A**) Cleavage efficiency of the different sgRNAs screened in wild-type hiPSCs relative to the parental (not cut) band. *HPRT* sgRNA was used as a positive control. The efficiency shown is a single experiment performed for quantification. (**B**) As in (A) but for TALEN1. TALEN against *HTR2A* was used as a positive control. (**C**) Representative gel of *HTR2A* and *PDE6C* TALENs cleavage assay in wild-type hiPSCs. hiPSCs were transfected with or without TALENs and PCR amplification of the desired locus was run in a 2% agarose gel for cleaved bands visualization. Red arrowheads indicate fragments with the expected size resulting from T7E1 cutting. Genomic DNA from hiPSCs transfected without TALENs (lane indicated with a minus sign) was used as a negative control, showing an intact parental band. (**D**) As in (**C**) but for *HPRT* and *PDE6C* sgRNA4. **(E)** ssODN designs for CRISPR/Cas9 and TALEN-mediated HDR repair in FRIMOi007-A. Notably, the ssODN template of CRISPR/Cas9 assay incorporates a Cas9-blocking mutation (purple arrowhead), which renders a synonymous amino acidic change. Red arrowhead indicates patient’s pathogenic variant. Nucleotide phosphorotioate modifications appear in orange.

In order to examine the cleavage efficiency of all these guides, we transfected them into wild-type hiPSC and detected DSBs through the endonuclease T7 I-mediated system. We achieved an overall DNA cleavage efficiency between 10% and 50% with the sgRNA/Cas9 system, similar to what has commonly been reported elsewhere [21] (Figure 2A), and of approximately 10% when using the TALEN1 pair (Figure 2B). Specifically, sgRNA1 and sgRNA3 cleave DNA with higher efficiency than sgRNA2 and sgRNA4 (Figure 2A), suggesting better DNA binding and Cas9 recruitment. However, all four sgRNAs and the TALEN1 mRNA pair demonstrated satisfactory cleavage efficiencies. In all cases, we confirmed the band size against the expected values according to the DSB site and the amplicon (Figure 2C,D).

These results show that all guides screened for targeting the c.1670G>A pathogenic variant effectively cut DNA at the desired *PDE6C* region. Thus, considering the distance, the absence of predicted exonic off-targets, and the cleavage efficiency (which was acceptable despite being lower than that of the sgRNA1 and sgRNA3), we selected sgRNA4 for the single-nucleotide CRISPR/Cas9-mediated assay. Notably, TALEN1 and sgRNA4 exhibit similar cleavage efficiencies (Figure 2A,B).

To achieve HDR-mediated repair, we designed specific ssODN templates for both technologies, considering several key parameters (detailed in the Materials and Methods). In addition to the specific disease-causing mutation correction (red in Figure 2E), the CRISPR ssODN incorporated a change in the PAM sequence to disrupt this motif (silent mutation).

To recapitulate, we chose sgRNA4 and TALEN1 guides (for CRISPR/Cas9 and TALENs, respectively) to precisely correct the *PDE6C* c.1670G>A pathogenic variant through ssODN-mediated repair in the FRIMOi007-Acell line. To promote HDR-mediated DNA editing, we used an HDR activator (L755507) together with an NHEJ inhibitor (M3814) (Figure 1A). 

### 2.3. Highly Efficient Genome Editing of a PDE6C Pathogenic Variant by CRISPR/Cas9 in hiPSCs

**Figure 3:**
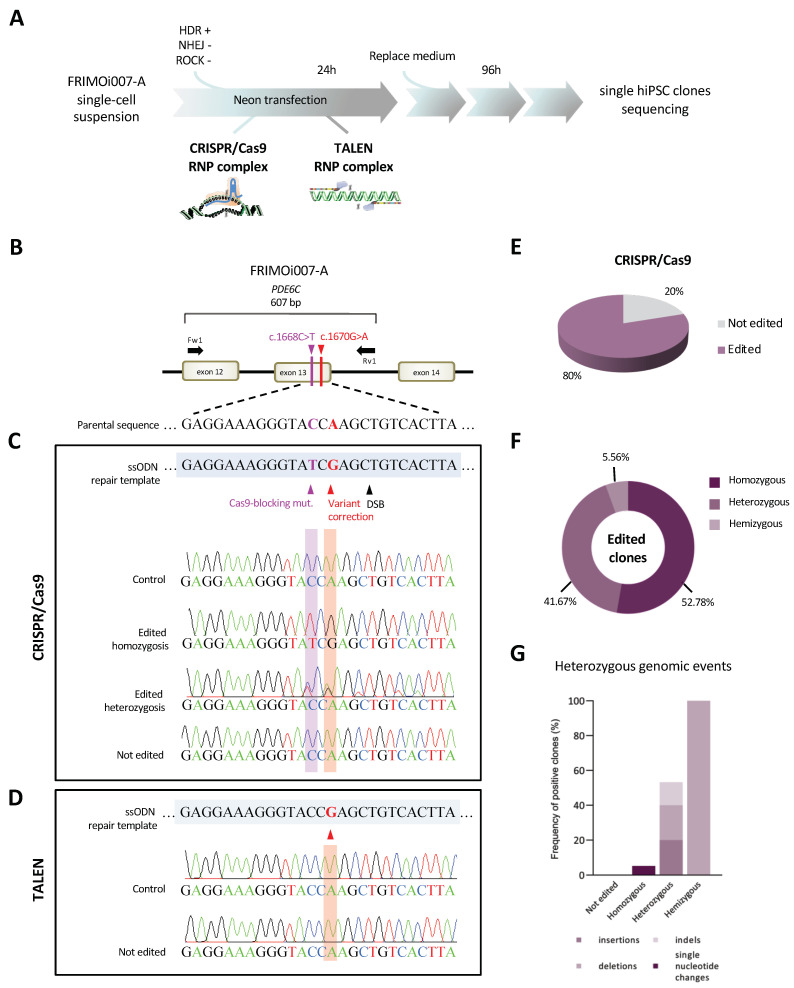
Highly efficient genome editing of a *PDE6C* pathogenic variant by CRISPR/Cas9 in hiPSCs and identification of on-target genomic defects. (**A**) Schematic representation of the assay conditions followed for CRISPR/Cas9 and TALEN-mediated genome editing in FRIMOi007-A. After 96h of cell culture, single colonies were picked and grown for analysis of the clones. The figure was partially generated using Servier Medical Art, provided by Servier, licensed under a Creative Commons Attribution 3.0 unported license. (**B**) FRIMOi007-A hiPSC clones were subjected to Sanger sequencing to analyze gene editing in the locus specified. Pathogenic variant is indicated in red and the Cas9-blocking mutation is in purple. (**C**) Representative captures from Sanger chromatograms of the control, edited in homozygosis, edited in heterozygosis, and unedited clones after CRISPR/Cas9 gene editing. Parental DNA sequence of FRIMOi007-A cells was used as a reference. Note that nucleotide changes are highlighted in purple and red as specified in scheme in (**B**). (**D**) As in (**C**), but for TALEN-mediated gene editing. (**E**) Quantification of screened clones after CRISPR/Cas9 gene editing according to variant correction. Results are represented as the percentage of positive clones out of total clones sequenced. (**F**) Percentage of edited clones in homo-, hemi-, or heterozygosis, out of all edited clones. (**G**) On-target genomic alteration quantifications in homo-, hetero-, and hemizygous edited clones and in unedited clones. Results are presented as the percentage of positive clones out of total clones from each type.

In order to correct the homozygous pathogenic variant in *PDE6C*, we subjected patient-derived hiPSCs to CRISPR/Cas9 and TALEN-mediated gene editing. FRIMOi007-A cells were transfected with ribonucleoprotein (RNP) complexes comprising either sgRNA or TALEN guides, the ssODN, and Cas9 HiFi protein (in the case of the CRISPR/Cas9 approach) (Figure 3A). hiPSCs transfected without sgRNA or TALEN were also obtained in parallel in each experiment as controls (control clones).

After electroporation and cell growth, we screened approximately fifty single isolated clones in the case of CRISPR/Cas9-treated hiPSCs and twenty-two viable clones obtained after TALEN transfection. All these single clones, together with control clones, were Sanger sequenced in the region of interest of *PDE6C* (Figure 3B). Accurate analysis of these sequences revealed that we had performed successful gene editing after the CRISPR/Cas9 system (Figure 3C), but not when using the TALEN approximation (Figure 3D). 

CRISPR/Cas9 yielded a significant efficiency in gene editing with 80% of the screened clones exhibiting *PDE6C* sequence correction (Figure 3E). In total, 36 clones out of 45 were found to be edited, from which 19 corrected the patient’s variants in homozygosis, 15 corrected the patient’s variants in heterozygosis, and 2 corrected the variants in hemizygosis (Figure 3C,F, and Table 2), indicating successful DSB and subsequent HDR-mediated repair. Surprisingly, only nine clones display no evidence of gene editing after sgRNA/Cas9 transfection (Figure 3E and Table 2). Notably, all control clones confirmed the presence of the patient variant, similar to the unedited ones (Figure 3C,D).

Bi-allelic amplification can be confirmed in heterozygous clones, but not in the case of homozygous clones, which lack SNPs or other genomic events, evidencing the amplification of both alleles [44]. Thereby, all homozygous clones were subjected to PCR amplification of the largest region on the *PDE6C* locus. Indels appeared, generally close to DSB; therefore, we decided to amplify near 1 kb around the cleavage site with Fw1 and Rv3 primers (Table S2 and Figure S1A). Gel analysis of PCR products revealed that most homozygous edited clones have a unique band with the expected size compared to thecontrol clone (Figure S1B). However, two of the clones initially classified as homozygous (according to previous Sanger results) exhibited two clearly separated bands, suggesting hemizygosis for the on-target region (Figure S1C). To further analyze these clones, both DNA fragments were purified by gel extraction and Sanger sequencing. The upper band with the expected size resulted in a corrected sequence—with variant and Cas9-silent mutation modifications—while the lower band showed a deletion close to the DSB of approximately either 340 bp or 200 bp, depending on the clone (Figure S1C).

Altogether, these data demonstrate that, in the majority of hiPSC clones (almost 53% of the edited clones), DSB and ssODN-mediated repair were properly performed in both alleles (Figure 3F). Significantly, we obtained an overall gene editing rate of 80%—clones in homo-, hetero-, and hemizygosis—with correction of the pathogenic variant in at least one of the alleles (Figure 3E).

### 2.4. Identification of Heterozygous on-Target Genomic Defects in Some Edited Clones

Gene editing tools have the disadvantage that they could undesirably generate genomic aberrations in on-target and off-target regions. To further analyze these edited clones, we screened them using different primers to genotype this *PDE6C* region (Figure S1A and Table S2). PCR and Sanger sequencing results confirmed that some of the heterozygous edited clones exhibit on-target abnormalities (three clones with insertions, three with deletions, and two carrying indels) and in all cases in heterozygosis (Table 2 and Figure 3G). The sizes of these alterations range from a few nucleotides to 150 bp, located close to the DSB.Remarkably, homozygous edited clones exhibited perfect sequence homology compared with the reference sequence, except for one that had incorporated a single-base change in heterozygosis (Figure 3C,G). Analysis of this nucleotide change showed that it generated a missense variant (c.1642T>C p.Trp548Arg) predicted by in silico analysis as damaging (Figure S1D). Despite the potential pathogenicity of this new mutation, it can be speculated that it would not trigger detrimental effects because it has been introduced in heterozygosity, similar to the other genomic aberrations, and this clone is homozygous for patients’ variant corrections. 

We then focused on the identification of which of the alleles was carrying the on-target defects in heterozygous corrected clones. Hence, we subjected these clones to PCR genotyping with two specific forward primers (Figure S1A and Table S2), aiming to discriminate between ssODN-corrected (primer named “Fw_sp_TCG”) and unedited (“Fw_sp_TCG”) alleles (detailed in Materials and Methods). In line with previous Sanger sequencing results, all screened clones exhibited one band for each of the two PCR reactions confirming heterozygosis (Figure S1E). Control and homozygous edited clones were run in parallel as amplification controls for both specific forward primers (Figure S1E). Strikingly, all heterozygous clones had an amplified band with the “Fw_sp_TCG” primer—for edited allele identification—with the expected wild-type size. However, the amplified fragment corresponding to unedited DNA revealed (i) the expected wild-type size, in the case of clones without on-target defects, or (ii) bigger or smaller bands sizes if insertions or deletions events occurred (lanes indicated with asterisks in Figure S1E). The conclusions derived from these analyses are two-fold: First, heterozygous edited clones with on-target genomic alterations were confirmed both by Sanger sequencing and PCR genotyping, and second, the allele without editing was harboring these aberrations. 

Collectively, these results suggest that the majority of edited clones have no on-target genomic anomalies and, importantly, none of them were found in homozygosis. The *PDE6C* genotyping results suggest that unedited alleles with on-target aberrations had not successfully corrected DNA DSB through HDR-mediated repair.

### 2.5. Single-Nucleotide Gene Editing Preserves hiPSCs Pluripotency and Renders no Genomic Alterations in Potential Off-Targets

**Figure 4:**
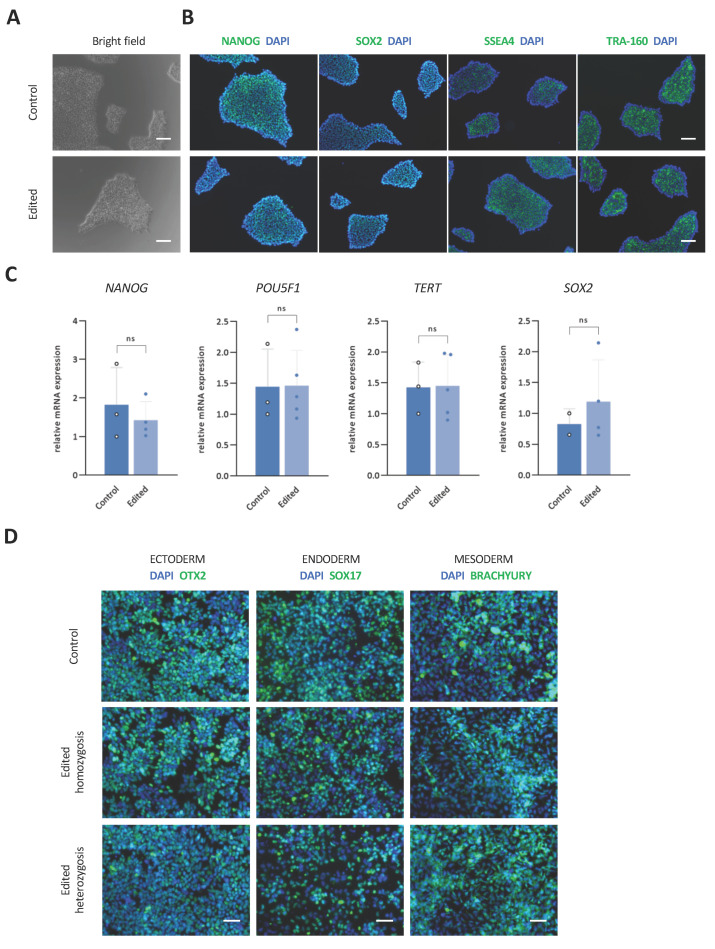
Single-nucleotide gene editing preserves hiPSCs’ pluripotency and renders no genomic alterations in potential off-targets. (**A**) Bright-field pictures of control and homozygous edited FRIMOi007-A clones showing colony morphology after CRISPR/Cas9. Scale bar represents 100 µm. (**B**) As in (**A**), but cells were stained to assess the expression of the pluripotency markers NANOG, SOX2, SSEA4, and TRA-160 by immunofluorescence with AF-488 (in green) and counterstained with DAPI. Representative captures are shown. Scale bar represents 100 µm. (**C**) Relative mRNA expression of pluripotency markers *NANOG*, *POU5F1*, *TERT*, and *SOX2* in control and homozygous edited clones. At least two clones were assessed for quantification. (**D**) Immunofluorescence assessment of OTX2, SOX17, and BRACHYURY differentiation markers in control and edited clones. Scale bar represents 50 µm.

To study whether hiPSCs had compromised cell growth and pluripotency after the CRISPR/Cas9 assay, corrected clones were cultured in parallel to control clones. Edited clones conserved hiPSC colony-like morphology in culture, and no differences in proliferation or morphology were observed during cell culture, compared with controls (Figure 4A). In addition, hiPSCs clones were assessed for pluripotency marker expression, at both mRNA and protein levels. Similar protein levels of NANOG, SOX2, SSEA4, and TRA-160 were observed by immunofluorescence in edited clones when compared with control hiPSCs, indicating the preservation of pluripotency (Figure 4B and Figure S2). Moreover, gene expression analysis of *NANOG*, *POU5F1*, *TERT*, and *SOX2* showed no significant differences between controls and edited clones (Figure 4C).

To assess whether gene editing compromised the ability of hiPSCs to differentiate into the three germ layers, we subjected edited clones to ectodermal, endodermal, and ectodermal lineages and analyzed them for the expression of OTX2, SOX17, and BRACHYURY differentiation markers, respectively. Immunofluorescence results showed no significant differences in the expression of these markers when compared with control clones (Figure 4D). 

**Figure 5:**
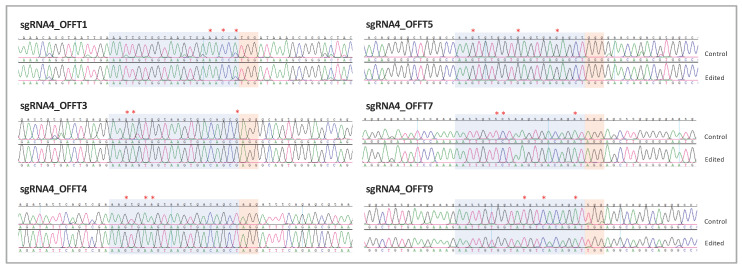
Edited clones exhibit no genomic alterations in potential off-targets. Representative captures of chromatograms showing Sanger sequencing reads of PCR products from the analyzed off-targets for *PDE6C* sgRNA4. On the top is the GRCh38 reference sequence; below are the results of one control and one homozygous edited clone for each off-target. Sequences homologous to the sgRNA and the adjacent PAM motif are highlighted. Red asterisks depict mismatches in sequence homology with the sgRNA.

Off-targets could generate undesired genomic alterations in other regions of the genome due to unintended DNA recognition; their detection could be difficult or ignored. In silico analysis of sgRNA4 predicted no off-target regions with fewer than three mismatches throughout the genome. Importantly, none of the off-targets with three mismatches corresponded to exonic regions (Table S1). Thus, we decided to analyze all intronic off-targets with three mismatches in all homozygous edited clones by Sanger sequencing (as specified in Table S1). We screened approximately 500 bp surrounding the six off-targets and checked the homologous sequence and the adjacent PAM motif for each one (Figure 5). Accurate analysis of these loci revealed that these edited clones had no genomic alterations (insertions, deletions, indels, or single-base modifications) in the ~500 bp homologous region analyzed, in comparison with control clones (Figure 5).

The results obtained in this study demonstrate that the use of CRISPR/Cas9-mediated gene editing is effective in precisely correcting single-nucleotide mutations without off-target effects. We obtained few undesired on-target genomic alterations but in all cases were in heterozygosis and in the unedited allele. These data indicate an overall gain of PDE6C function due to the high percentage of clones with the corrected pathogenic variant, both in homo- and heterozygosis, and highlight the use of CRISPR/Cas9 technology as a potential tool for the treatment of ACHM.

## 3. Discussion

Inherited retinal dystrophies are a broad group of eye disorders affecting diverse cell types in the retina that trigger visual complications and, in most cases, blindness. Among them is ACHM, a rare disease that completely eliminates color vision, accompanied by other eye-related problems. Despite their severe phenotypes conditioning individuals’ normal life, no cures are available for any IRDs. Moreover, these pathologies have a genetic origin and are heritable, making genetic diagnoses and counseling of vital importance for their management. Thus, it is urgent to find treatments for these genetic eye disorders. 

Gene editing has arisen as a promising potential therapeutic strategy for future medicine [40,41]. In fact, virtually all genes can be modified by ever-developing gene editing tools [45]. Achromatopsia is caused by mutations in six reported genes, which, in the vast majority, comprise single-nucleotide changes generating missense and truncating mutations or alterations in splicing patterns [4,5,6,46]. Therefore, ssODN-mediated gene editing for the precise correction of these mutations seems appropriate and could be a promising tool for the definitive reversion of this disorder.

Base editing and prime editing technologies are also powerful tools to perform all transition and transversion mutations, as well as small insertions and deletion changes without DSBs and repair templates [45]. However, their efficiency, accuracy, and targetability remain controversial [45,47]. In addition, off-target evaluation is difficult, and many studies have reported nucleotide mutations outside the off-targets predicted in silico [47,48,49,50]. In this manuscript, we show the efficient correction of a missense *PDE6C* variant causing ACHM by CRISPR/Cas9 through HDR-mediated DNA repair. We obtained a significant number of properly edited clones—almost 80% of screened clones, both in homo- and heterozygosis—which could potentially restore functional PDE6C protein, due to the recessive behavior of the associated phenotype. Remarkably, the majority of clones exhibiting evidence of gene editing were homozygous, with perfect on-target homologous sequences (Figure 3C,F). 

Although the CRISPR/Cas system is the most widely used genome editing strategy, TALENs are more effective in HDR repair [51,52]. However, CRISPR/Cas9 technology is more efficient, powerful, and flexible [53,54]. Unfortunately, we have not been able to obtain corrected clones through TALENs-mediated gene editing. It is worth noting that hiPSCs recovery and viability after TALEN transfection was significantly compromised compared with hiPSCs treated with sgRNA/Cas9. Hence, the low number of viable clones could hamper the screening of corrected clones. Notably, the cleavage efficiency between sgRNA4 and TALENs in wild-type hiPSCs was similar, indicating that DSB was not the problem in gene editing when comparing these two technologies.

Despite the effectiveness of the CRISPR/Cas9 system, many parameters and considerations need to be addressed for improving assay design. A balance between ssODN, sgRNA, and Cas9 amounts transfected in cells has to be finely tuned to achieve efficient HDR repair. Nonetheless, the number of hiPSCs that escape from this template-mediated repair or incorporate errors is, in some cases, abundant. NHEJ is the main method by which cells repair CRISPR/Cas9-derived DSBs [55]. It is a highly effective but also error-prone mechanism and is considered, together with MMEJ, to be the major way of introducing undesired indels at DSBs [26,55]. On the other hand, HDR activators and NHEJ inhibitors could also be modulated to improve HDR-repaired hiPSCs [26,45]. Notably, an increase in HDR efficiency has been reported when using ssODNs as repair templates for single-nucleotide modifications [56]. An increase in ssODN concentrations could increase template-mediated repair but may be detrimental to on-target or off-target effects.

On-target anomalies and off-targets are two of the main problems of the CRISPR/Cas9 system [41,44,45,57]. Strikingly, on-target effects have been reported in the literature in up to 40% of hiPSC clones after CRISPR editing [57]. However, in recent years, many approximations have arisen trying to address them [57,58,59]. sgRNA4, selected for gene editing in our study, has no off-targets with fewer than three mismatches in sequence homology throughout the genome (Table S1), which is one of the main parameters to take into account in sgRNA designs. Importantly, all homozygous corrected clones except one (which incorporated a single-nucleotide change) exhibited no undesired aberrations, either in the edited region or in off-targets, demonstrating the effectiveness of the CRISPR/Cas9 system. These results suggest a single and successful cycle of DNA DSB and HDR repair due to the absence of on-target abnormalities and the inclusion of Cas9-blocking mutation. Moreover, on-target genomic anomalies were not observed in unedited clones, suggesting a lack of effective electroporation in some hiPSCs, the absence of sgRNA binding, or deficiencies in Cas9 recruitment. 

Falsely corrected clones due to incomplete genotyping are one of the main issues to be addressed after gene editing and could be a problem for downstream studies [44,57]. Due to genomic aberrations, sequencing results might lead to the misclassification of clones because the affected allele is not amplified, and those that are apparently homozygous may, in fact, be hemizygous [44]. In our study, we only detected approximately 25% of the edited clones displaying on-target heterozygous abnormalities and two hemizygous clones (Figure 3G). Remarkably, all on-target defects were in the unedited allele suggesting the lack of proper HDR-mediated repair. It is worth noting that the correction of a homozygous pathogenic variant disables the possibility to have the Cas9-silent mutation in a heterozygous state demonstrating a bi-allelic dose unless another heterozygous marker (e.g., SNP) in the vicinity exists [44,57]. We, similar to other authors, have found insertions and deletions in on-target regions [29,44,57,58,60]. Notably, the size of the indels described in our results ranged from a few to approximately 300 nucleotides. It can be speculated that large indels could arise after gene editing and were thus not detected through the genotyping performed here. However, we did not use the Cas9 overexpression strategy, which has been associated with large insertions of plasmid DNA [61,62]. Moreover, the RNP-based transfection method followed in this study has been reported to generate a higher number of properly edited clones, as well as fewer unwanted monoallelic editing events [30,44,61]. 

Several phase I/II clinical trials for gene therapy using AAV to deliver *CNGA3* and *CNGB3* are currently in process for the treatment of ACHM [63].Genome editing in hiPSCs represents an enormous tool for disease investigation and molecular and cellular research avoiding the use of viral vectors to introduce exogenous material, as occurs with other therapeutic approximations.Additionally, gene editing enables the permanent correction of pathogenic variants in a patient’s hiPSCs, which are a potentially unlimited cellular source for autologous cellular therapy. Moreover, the accessibility and easy monitorization of the eye make IRDs good candidates for future cell and gene therapy applications [64]. 

Despite the efficacy of CRISPR/Cas9, many concerns have yet to be faced before clinical applications. Some of the main limitations of gene editing are its in vivo delivery, editing efficiency, and accuracy. Nevertheless, successful delivery in the treatment of Leber congenital amaurosis type 10 through direct subretinal injection has recently been reported [31,65]. In addition, the accessible and easy assessment of off-target defects remains elusive, especially in vivo. Other issues regarding logistical limitations or difficulties in manufacturing edited cells are also problems to overcome for future CRISPR-based medicine [45].

The results obtained in this manuscript show, for the first time, the efficient correction of a homozygous pathogenic variant in *PDE6C* causing ACHM in patient-derived iPSCs by CRISPR/Cas9/ssODN-mediated gene editing. These data indicate an overall gain of PDE6C function due to the high percentage of clones with corrected pathogenic variants, both in homo- and heterozygosis. All edited clones in this study could potentially generate a more functional PDE6C protein than that found in the patient, thus alleviating, at least in part, the symptomatology. Research conducted on these therapeutic approaches is crucial for the advancement of future translational and personalized medicine in IRDs, thus significantly contributing to the development of potential ACHM treatment.

## 4. Materials and Methods

### 4.1. hiPSC Culture and Transfection

The human iPS cell line (FRIMOi007-A) derived from an ACHM patient (Fi22/02) carrying a homozygous mutation in *PDE6C* was obtained, as described in Domingo-Prim, J. et al. [66]. For some experiments, wild-type hiPSCs were also used, which were acquired from a patient who did not present with an ophthalmologic disease or any genetic variants related to ACHM. hiPSCs colonies were maintained in StemFlex medium (Thermo Fisher Scientific, Waltham, MA, USA) and cultured on Matrigel-coated dishes (Merck, Bedford, MA, USA). To obtain hiPS single-cell suspensions, hiPS colonies were detached with TrypLE (Thermo Fisher Scientific, Waltham, MA, USA), centrifuged, and counted before Neon-mediated transfection (Thermo Fisher Scientific, Waltham, MA, USA). For hiPSC differentiation, clones were subjected to mesodermal, ectodermal, and endodermal lineages and analyzed with the Human Pluripotent Stem Cell Functional Identification Kit (R&D Systems, MN, USA), according to the manufacturer’s instructions.

### 4.2. sgRNAs, TALENs and ssODNs Design

sgRNAs and TALENs were designed using the Invitrogen™ TrueDesign™ Genome Editor (Thermo Fisher Scientific, Waltham, MA, USA), as detailed in Table 1. sgRNAs were selected according to their predicted efficiency and the lowest number of potential off-targets. *HPRT* sgRNA and *HTR2A* TALEN pairs, used as positive controls for CRISPR and TALEN cleavage assessments, respectively, were purchased from Thermo Fisher Scientific. ssODNs designs were performed according to the following premises: The cutting site was centered and the ssODN was designed with a total length of between 75 and 85 nucleotides, ensuring 30–35-nucleotide lengths of the left and right arms with perfect sequence homology. Phosphorothioate nucleotide modifications were added to the ends of the ssODN (as shown in orange in Figure 2E) to increase stability [60,67] and were synthesized with the PAGE purification method. The ssODN sequence is specified in Figure 2E. PAM sequence modification was incorporated into the ssODN repair template with the mutation in the second or third nucleotide of the PAM motif (NGG) (shown in purple in Figure 2E) to avoid re-cutting of the target DNA by Cas9 after HDR repair [44,67]. Conservation of the reading frame, amino acid change, splicing pattern, and SNP prevalence of the nucleotide modification was consulted with ALAMUT software (version 1.4, Sophia Genetics, Switzerland) according to the following predictors: Splice Site Analysis (SFF), MaxEnt, Splice Site Prediction by Neural Network (NNSPLICE), and GeneSplicer. Nucleotide changes were analyzed with PhyloP and the UCSC Genome Browser.

### 4.3. Genomic Cleavage Detection Assay

For the detection of genomic DNA cleavage by CRISPR/Cas9 and TALEN approaches, we used the GeneArt Genomic Cleavage Detection Kit (Thermo Fisher Scientific, Waltham, MA, USA), according to the manufacturer’s instructions. Briefly, hiPSCs were transfected with sgRNA or TALEN, and four days later, PCR amplification of the desired locus was performed and run in a gel to ensure a single band. Next, the PCR product was subjected to several rounds of denaturation and re-annealing to generate mismatches, which were detected and cleaved by using the Detection Enzyme. The results were visualized by gel electrophoresis with iBrightCL1000 (Thermo Fisher Scientific, Waltham, MA, USA) and band intensity quantification was performed with iBright^TM^ Analysis Software (Thermo Fisher Scientific, Waltham, MA, USA), according to the manufacturer’s instructions. Band intensity quantification was correlated with the Cas9 or TALEN activity.

### 4.4. Human iPSC CRISPR/Cas9 and TALEN-Mediated Genome Editing

For genome editing, 1 × 10^5^ iPSCs were electroporated with 10 pmols sgRNA or 100 ng TALENs, 15 pmols ssODN, and 10 pmols High Fidelity Cas9 protein (in the case of the CRISPR/Cas9 assay) (Thermo Fisher Scientific, Waltham, MA, USA). In parallel, hiPSCs without sgRNA and TALENs were also transfected as controls. Electroporation was performed with the Neon transfection system (Thermo Fisher Scientific, Waltham, MA, USA) with optimal electroporation conditions found to work better in our cells: Two pulses of 1200 V and 20 ms. Immediately after electroporation, hiPSCs were seeded on Matrigel-coated dishes and cultured in the StemFlex medium supplemented with 10 µM of the ROCK inhibitor (Merck, Bedford, MA, USA), 10 µM of the HDR activator L755507 (Merck, Bedford, MA, USA) [68], and 0.5 µM of the NHEJ inhibitor M3814 (Selleckchem, USA) [26] for 24h. Then, the cell culture medium was replaced by a fresh StemFlex medium, and cells were cultured until colonies formed from the single-cell suspension. When colonies were grown but were still small enough to ensure individual clones, over fifty clones were picked and cultured, and more than twenty hiPS cells were subjected to TALEN transfection. After approximately one week, individual clones were collected for culture and subjected to genotyping analysis by Sanger sequencing (Macrogen, Spain). Edited and control clones were further expanded and sequenced again to confirm the desired genotype.

### 4.5. PCR Amplification, Gel Extraction, Sanger Sequencing and Data Analysis

PCR amplification of the desired genomic region was performed and run in a gel to ensure a single DNA band and negative control. PCR products were purified using 96-well Acroprep Advance plates (Pall Corporation, Ann Arbor, MI, USA) with a vacuum manifold (Pall Corporation) and Sanger-sequenced with forward and reverse primers in Macrogen Spain. For *PDE6C* genotyping, we designed two forward primers sharing the same sequence but differing only in two of the last three 3’ nucleotides. Specifically, these nucleotides corresponded to the Cas9-blocking mutation and to variant correction. A forward primer ending with TCG—named Fw_sp_TCG—was designed to amplify DNA corrected with the ssODN (thus incorporating both nucleotide modifications), and a forward primer ending with CCA—referred to as Fw_sp_CCA—was used to identify unedited alleles. All primer sequences used in this study are detailed in Tables S2 and S3. Sanger sequencing results were downloaded from the manufacturer’s platform, and data were aligned and analyzed. Sequences were assembled with the reference locus sequence according to the GRCh38 human genome. When needed, DNA fragments were gel extracted and purified with GeneAllExpin Combo GP (GeneAll, Seoul, Korea) and the GeneJET Gel Extraction Kit (Thermo Fisher Scientific, Waltham, MA, USA), according to the manufacturer’s instructions. 

Missense mutations were analyzed with the ENSEMBL Variant Effect predictor (VEP) [69], which provides results from a range of algorithms to assess the potential pathogenicity of a variant. Predictors used by VEP are LRT, MutationTaster, FATHMM, PROVEAN, MetaSVM, MetaLR, MetaRNN, PRIMATEAI, DEOGEN2, BayesDel_addAF, ClinPred, fathmm_MFL_coding, fathmm_XF_coding, SIFT, Polyphen, and Loftool.

### 4.6. Off-Target Prediction and Analysis

Off-target prediction was performed using the online tool Cas-OFFinder [70]. A maximum of three mismatches were allowed for the algorithm to run the prediction. To assess potential off-target alterations, all intronic regions were covered with Sanger sequencing. Intergenic regions were not analyzed in this screening. PCR amplification of the selected off-target regions (specified in Table S1) was performed in 3 control clones and 13 properly edited clones, which were then subjected to Sanger sequencing.

### 4.7. Immunofluorescence Staining

For the immunofluorescence analysis of pluripotency markers, hiPSC clones were seeded on Matrigel-coated ibidi slides (ibidiGmbH, Germany) and cultured in the StemFlex medium. When colonies were formed, ibidi slides were fixed in 4% paraformaldehyde (Thermo Fisher Scientific, Waltham, MA, USA) for 15 min at room temperature. Next, cells were permeabilized with 0.25% Triton X-100 in PBS and incubated for 1 h in a blocking solution (5% FBS, 4% BSA, and 0.5% Tween in PBS) at room temperature. hiPSC clones were then incubated overnight at 4 °C with NANOG (D73G4, Cell Signaling Technology, MA, USA), SOX2-AlexaFluor488 (E-4, Santa Cruz Biotechnology, TX, USA), SSEA4-AlexaFluor488 (BD Pharmingen), or TRA-160-AlexaFluor488 (BD Pharmingen) antibodies. Anti-rabbit AlexaFluor-488 (Invitrogen, MA, USA) conjugated secondary antibody was used for NANOG staining. Immunofluorescence visualization and pictures were performed with a Zeiss Axiovert and Axiocam 503 mono (CarlZeissInc., North America). Fluorescence pictures were processed using ImageJ software.

### 4.8. RNA Extraction and Quantitative Real-Time PCR

To assess the gene expression of pluripotency markers, RNA from hiPSCs was extracted using Trizol (Thermo Fisher Scientific, Waltham, MA, USA), according to the manufacturer’s instructions. cDNA was obtained with the Transcriptor First Strand cDNA Synthesis Kit (Roche Diagnostics, Basel, Switzerland). Real-time PCR was performed in QuantStudio™ (Thermo Fisher Scientific, Waltham, MA, USA) using TaqMan probes (Thermo Fisher Scientific, Waltham, MA, USA). 

### 4.9. Statistical Analysis

When needed, statistical analysis of the data was performed using Prism 9.3.1 (GraphPad Software, La Jolla, California, USA). Statistical significance was assessed with a non-parametric Mann–Whitney U-test to compare control and edited clones. Bar graphs in Figure 4C show the mean and standard error of the mean. Non-significance (ns) was set at a value of *p* > 0.05. Bar graphs in Figure 2 and Figure 3 represent individual values.

## Figures and Tables

**Table 1:** sgRNA and TALEN guides designed for targeting *PDE6C* c.1670G>A variant.

Sequence ID	Technology	Sequence	PAM	Strand	Distance	Off-Targets (3 Mismatches)	Exonic Off-Targets
sgRNA1	CRISPR	CTTACCACAATTGGCGGCAT	GGG	+	+25	1	0
sgRNA2	CRISPR	TTGGCGGCATGGGTTCAACG	TGG	+	+35	2	1
sgRNA3	CRISPR	CAATTGGCGGCATGGGTTCA	TGG	-	+18	3	1
sgRNA4	CRISPR	AGCTGTCACTTACCACAATT	CGG	-	+3	10	0
TALEN1	TALEN	TGTACACTGTGAGGAAAG, TGCCGCCAATTGTGGTAA	--	+/−	--	--	--

**Table 2:** Results of clones genotyping after gene editing.

					Genomic Events in Heterozygosis		
Technology	Clones Screened	Variant Correction		Insertions	Deletions	Indels	Single-Base Changes
CRISPR/Cas9	45	unedited	9	0	0	0	0
		homozygosis	19	0	0	0	1
		heterozygosis	15	3	3	2	0
		hemizygosis	2	0	2	0	0
TALEN	22	0		0	0	0	0

## Data Availability

The data presented in this study are available in the article and supplementary material. Any other data related to the manuscript is available upon reasonable request.

## Supplementary Materials

The following supporting information can be downloaded at: https://www.mdpi.com/article/10.3390/ijms24043655/s1, Figure S1: Identification of heterozygous on-target genomic defects in some edited clones but virtually not affecting PDE6C function; Figure S2: Single-nucleotide gene editing preserves hiPSCs pluripotency; Table S1: Off-targets list for each sgRNA; Table S2: List of primers used for *PDE6C* genotyping; Table S3: List of primers used for genotyping.
