# Supplementary material for: High-Efficiency CRISPR/Cas9-Mediated Correction of a Homozygous Mutation in Achromatopsia-Patient-Derived iPSCs
Source: Int J Mol Sci. 2023 Feb 11;24(4):3655. doi: 10.3390/ijms24043655 (PMC9964936; doi:10.3390/ijms24043655)
Supplement: Supplementary file 1 [file ijms-24-03655-s001.zip › ijms-2156258-supplementary.pdf]

<u>Supplemental Material for</u>:

High-efficiency CRISPR/Cas9-mediated correction of a homozygous mutation in achromatopsia-patient-derived iPSCs

Figure S1

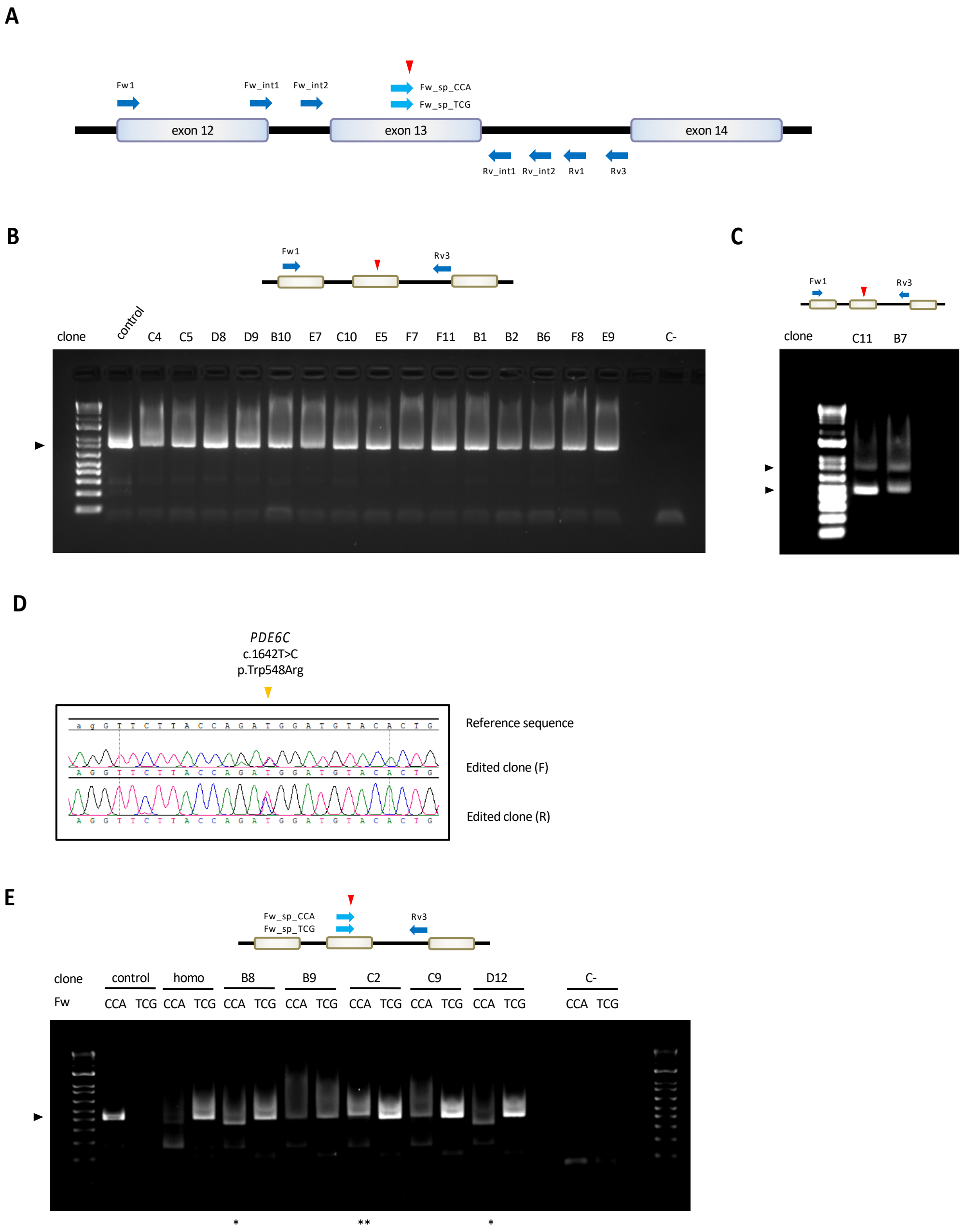

Figure S1. Identification of heterozygous on-target genomic defects in some edited clones but virtually not affecting PDE6C function. **(A)** Schematic representation of primers designed for genotyping *PDE6C* locus surrounding the c.1670G>A pathogenic variant. The forward primers appear above and below are the reverse ones. Primers sequences are detailed in Table S3. **(B)** Representative agarose gel of PCR products from one control and some homozygous edited clones amplified with Fw1 and Rv3 primers showing unique DNA band. **(C)** As in (B) but with the two hemizygous clones carrying the on-target deletion in one allele. **(D)** Sanger sequencing results of the edited clone displaying a single-nucleotide modification in heterozygosis. Nucleotide change is indicated in yellow. Sequencing was done with forward (F) and reverse (R) primers. **(E)** Heterozygous edited clones with on-target abnormalities were amplified with specific forward primers to identify the allele harboring the indels. PCR products from representative clones were run in a gel together with a control and homozygous edited clones ("homo"). "CCA" indicates PCR performed with the specific primer Fw_sp_CCA and "TCG" with specific primer Fw_sp_TCG. Asterisks depict clones with deletions (single asterisk) or insertions (double asterisk), according to band size compared with the wild-type.

Figure S2

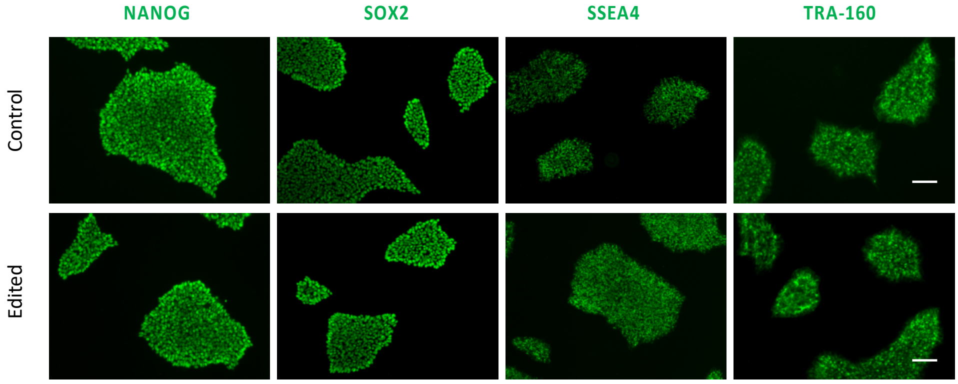

Figure S2. Single-nucleotide gene editing preserves hiPSCs pluripotency. Single staining pictures of Figure 4B, for the expression of NANOG, SOX2, SSEA4 and TRA-160. Scale bar represents 100 μm.

Table S1. Off-targets list for each sgRNA.

Off-target ID	DNA sequence	Chromosome	Position	Strand	Mismatches	Locus type
sgRNA1_OFFT1	CTgACCACAATTaGgGGCATAGG	chr1	110917468	+	3	Intergenic
sgRNA2_OFFT1	cTGGCGGCAcGGcTTCAACGTGG	chr5	149895208	-	3	*PDE6A* exon 13
sgRNA2_OFFT2	TTGGaGGCATGaGTTCAcCGGGG	chr12	132872780	-	3	*CHFR* intron 3
sgRNA3_OFFT1	TGAACCCgTGCtGCCcATTGTGG	chr3	56079354	-	3	*ERC2* intron 7
sgRNA3_OFFT2	TGAAgCCgTGCCGCCAgTTGTGG	chr5	149895214	+	3	*PDE6A* exon 13
sgRNA3_OFFT3	TGAACCCATGCCGgCAATatGGG	chr19	46953664	-	3	*ARGGAP35* intron 3
sgRNA4_OFFT1	AATTGTGGTAAGTGAaAcCaTGG	chr3	15410656	+	3	*METTL6* intron 6
sgRNA4_OFFT2	AcTTGgGGTAAGgGACAGCTTGG	chr3	79893056	+	3	Intergenic
sgRNA4_OFFT3	AAgaGTGGTAAGTGACAGCgAGG	chr3	196287515	-	3	*PCYT1A* intron 1
sgRNA4_OFFT4	AAgTGaaGTAAGTGACAGCTAGG	chr10	6563173	+	3	*PRKCQ* intron 1
sgRNA4_OFFT5	AAgTGTGGTgAGTGAgAGCTGGG	chr11	66916128	-	3	*PC* intron 3
sgRNA4_OFFT6	AATTGTGGTAAGTGACAtaaAGG	chr11	98091538	+	3	Intergenic
sgRNA4_OFFT7	AATTGTtcTAAGTGACAGaTAGG	chr12	66469809	-	3	*GRIP1* intron 7
sgRNA4_OFFT8	AAgTGTGGTcAGTtACAGCTTGG	chr17	76789775	-	3	Intergenic
sgRNA4_OFFT9	AATTGTGGTAtGTcACAGaTTGG	chr19	58192282	+	3	*ZNF274* intron 1
sgRNA4_OFFT10	AATTGTGGTAAGacAgAGCTAGG	chrX	15101360	-	3	Intergenic

[a]Mismatched nucleotides are indicated in minus
[b]Off-targets analyzed by Sanger sequencing

Table S2. List of primers used for *PDE6C* genotyping.

Primer ID	Location	Sequence	Distancefrom target (bp)
Fw1	exon 12	CGAATTCCGCTTCAGTGACTTC	-382
Rv1	intron 13	CGGTGCTCAAGGATACTGGGAC	+224
Fw_int1	exon 12 + intron 12	GAAATTCAAAGTACCTGTAGAGG	-295
Rv_int1	intron 13	CATACCTATTTCTCATCATGCTC	+194
Fw_int2	intron 12	TGTGGCTCTGCTTCACTGATG	-237
Rv_int2	intron 13	AGCGGAGAGGGGTTTGTGC	+140
Rv3	intron 13	ACCTCTATACCAAGTGCAGCC	+379
Fw_sp_CCA	exon 13	CACTGTGAGGAAAGGGTACCA	-21
Fw_sp_TCG	exon 13	CACTGTGAGGAAAGGGTATCG	-21

Table S3. List of primers used for genotyping.

Gene	Primer Forward	Primer Reverse
HTR2A	AGAAAATTACACAGCAATAAAATATAGCGG	CCAATATTAATATGTAGCAAAAAGAGGGAG
HPRT	ACATCAGCAGCTGTTCTG	GGCTGAAAGGAGAGAACT
OFFT#1	AACCCAAGAGGCGGAGACTG	TCCACCTTCCAGGCTCAAGC
OFFT#3	ACTACTGTGTCAGGAGTCGTG	AGTGCCGTCGCAGCCCCG
OFFT#4	AGTAGAGCTCAGTCATAGTAGG	TTTCAAGGTGGGCTTCCTTGG
OFFT#5	AGGAGGTGAAGACCAGCCTG	AATAGCCACTGCCTCTTTCGG
OFFT#7	TTCTGGTCTGTGTGATGGAGG	TAGCAAGATTCTATCATAGTAAGG
OFFT#9	AGACAGCTTCTAGGCATGGAG	ACTCACCTAGGTCCACTGAG
